# The Importance of Rapid Influenza Testing in Pediatric Primary Care: Experience During Three Consecutive Influenza Seasons (2016–2019) in Barcelona (Catalonia, Spain)

**DOI:** 10.3389/fped.2020.00565

**Published:** 2020-10-08

**Authors:** Diego van Esso, Monica Vilà, Cristina Andrés, Sheila Iglesias, Josep Ferrer, Lucia Losada, Gemma Ricos, Lorena Sanchez, Patricia Morera, Mar Pérez, Mª Angeles Ferrandez, Esther Hernando, Carlos Rodrigo, Tomàs Pumarola, Andrés Antón

**Affiliations:** ^1^Primary Care Health Service SAP Muntanya, Catalan Institute of Health, Barcelona, Spain; ^2^Primary Care Carmel, Catalan Institute of Health, Barcelona, Spain; ^3^Respiratory Virus Unit, Microbiology Department, Vall d'Hebron Research Institute, Vall d'Hebron University Hospital, Autonomous University of Barcelona, Barcelona, Spain; ^4^Primary Care CAPI Casernes, Catalan Institute of Health, Barcelona, Spain; ^5^Primary Care Roquetes-Canteres, Catalan Institute of Health, Barcelona, Spain; ^6^Primary Care LP Drassanes, Catalan Institute of Health, Barcelona, Spain; ^7^Primary Care LP La Marina, Catalan Institute of Health, Barcelona, Spain; ^8^Primary Care LP Pare Claret, Catalan Institute of Health, Barcelona, Spain; ^9^Primary Care Ciutat Meridiana, Catalan Institute of Health, Barcelona, Spain; ^10^Primary Care LP Rio de Janeiro, Catalan Institute of Health, Barcelona, Spain; ^11^Department of Paediatrics, Vall d'Hebron University Hospital, Barcelona, Spain; ^12^School of Medicine Germans Trias i Pujol University Hospital, Autonomous University of Barcelona, Barcelona, Spain

**Keywords:** influenza, primary care, children, rapid influenza diagnostic test (RIDT), point of care test (POCT), pediatrics, respiratory viral infection

## Abstract

Clinical diagnosis of influenza has low sensitivity in infants and children. Signs and symptoms are non-specific and similar to those of other respiratory viruses. Rapid influenza diagnostic tests (RIDTs) with adequate sensitivity and specificity used at the point of care can be useful for an etiologic diagnosis of influenza in primary care. This should have an impact on better management of these patients. We conducted a study during three consecutive influenza seasons (2016–2017, 2017–2018, and 2018–2019) in pediatric primary care settings collecting data from influenza point-of-care tests (POCTs)-confirmed ≤ 6-year-old patients. During the first two influenza seasons, antibiotic prescriptions and additional visits from influenza POCT-confirmed patients (Group_1) were compared to patients with influenza-like illness (ILI) (Group_2), or fever (2016 ICD-10 code R50) with no other signs of influenza (Group_3). Group_1 had 0.19 (2016–2017) and 0.23 (2017–2018) additional visits compared to 0.48 (2016–2017) and 0.49 (2017–2018) Group_2 *p* < 0.001 and 1.01 (2016–2017) and 0.80 (2017–2018) Group_3 *p* < 0.001. Antibiotic prescription was lower in Group_1 (10.2%) vs. Group_3 (17.2%) *p* < 0.002, difference statistically significant only for the 2017–2018 season. During the third season (2018–2019), RIDTs results were transmitted in real time to the reference laboratory ia the cloud, which strengthens the monitoring of circulating influenza viruses in the community. In our experience, the use of POCTs has a great potential in primary care specially in infants and young children in which the diagnosis maybe missed due to non-specific signs and symptoms.

## Introduction

Influenza virus is an important cause of respiratory illness among infants and children. The burden of influenza in pediatric primary care is not well-known as patients are seldom investigated for virologic evidence of influenza. Morbidity is highest in children with attack rates of 30% or more (1, 2). In day care attendees rates up to 50% have been reported (1). Cromer et al. (3) estimated the burden of influenza in England and reported that the highest influenza admission rates are found in children <5 years (1.9/1,000) and that infants <6 months had the highest consultation rates. Poehling et al. (4) reported in USA a rate of influenza hospitalization of 0.4 per 1,000 children and 50 clinic visits attributable to influenza per 1,000 children during the 2002–2003 season, and 1,5 hospitalizations and 97 clinic visits per 1,000 children during the 2003–2004 season showing a great variability of the burden of disease between seasons, a fact that may be partially attributed to the specific circulating viruses in each season (1, 2). Neuzil et al. (5), in a 25-year-prospective study also confirmed that influenza is a common infection in healthy children <5 years with a high burden of influenza related consultations. Mortality rates caused by seasonal influenza are generally low in children (<1/100,000 people per year) (1). Children play a key role in the transmission of influenza as they act spreading the disease in the community. Morbidity in this age group is higher than in adults due to limited pre-existing immunity, close contact in schools or day care, hygienic habits and due to their higher viral load and persistent shedding (6).

Clinical diagnosis of influenza is difficult in infants and young children because of non-specific signs and symptoms. Fever, cough and nasal congestion are symptoms of most viral respiratory infections. In their study, Peltola et al. (7) reported that both sensitivity and positive predictive value of clinical diagnosis were low (38 and 32%, respectively). Myalgia, headache, and malaise, useful features for a clinical diagnosis in older children and adults, are not useful in infants and young children unable to reliably describe them (8).

In the absence of a confirmed influenza microbiologic diagnosis using point-of-care tests (POCTs) in primary care settings, infants and young children are more frequently attended in hospitals, submitted to laboratory tests, and receive unnecessary antibiotics (9). The percentage of patients with an unspecific diagnosis of viral infection who receive antibiotic treatment is high but varies widely depending on the country's antibiotic policy and the site in which the patient is diagnosed (emergencies, primary care, or hospital). The consequence of an unspecific diagnosis is that patients often need to be reassessed if the symptoms persist, increasing the burden on the healthcare system.

The primary objectives of this study, during the first two consecutive influenza seasons (2016–2017 and 2017–2018), were the comparison of antibiotic prescriptions and the number of additional consultations to healthcare centers 10 days after diagnosis between children with POCT-confirmed influenza in the primary care office with those diagnosed of influenza like illness (ILI) or fever when no other signs of influenza were apparent. During the third consecutive season (2018–2019), weekly results of RIDTs in children ≤ 6 years reported from primary care were compared with influenza cases of the reference hospital and the sentinel influenza surveillance system of Catalonia (Spain). Our secondary objectives were to (i) describe and compare the clinical signs and symptoms between positive and negative influenza patients, as well as between influenza A (IAV) and B (IBV) virus infections, (ii) compare the performance of the Sofia test to a real-time reverse transcription polymerase chain reaction (RT-PCR) analysis, which is the current gold standard.

## Materials and Methods

We conducted a descriptive, prospective, longitudinal study during three consecutive influenza seasons (2016–2017, 2017–2018, and 2018–2019) in four (first season) and seven (second and third seasons) primary care centers of Barcelona, involving a total of 22 pediatricians. This study was reviewed and approved by the ethics committee of IDIAP Jordi Gol (P16/135) and conducted in compliance with the Declaration of Helsinki and the principles of Good Clinical Practices.

During the first two seasons, eligible participants included children ≤ 6 years presenting either (i) axillary temperature ≥38°C or (ii) axillary temperature between 37.2 and 38.0°C with rhinorrhoea, nasal congestion, or cough within 72 h from symptom onset. No exclusion criteria were established. Nasopharyngeal swabs from enrolled patients were collected in a tube with viral transport medium. Patients were asked to complete a diary for 10 days to assess clinical symptoms, antibiotic treatment taken during this period, and the additional visits at any healthcare service. This information was collected by a phone call, maximum 3 attempts, starting the first working day after the 10-days period.

Before enrolment in the study for the first two seasons, written and informed consent was obtained from parents or legal guardians concerning sample collection and follow-up. Eligible patients who were not willing to sign informed consent were not tested. During the third season, no clinical data were collected, and no follow-up or microbiological comparison with a molecular assay were done. Therefore, informed consent was not required. Data of the third season were collected in the seven primary care centers and transmitted in real time through a cloud system to the microbiology laboratory and were compared weekly with data of hospital patients and cases reported by the sentinel respiratory virus surveillance system of Catalonia.

During the first season (2016–2017), respiratory specimens were tested in parallel using two rapid tests: Sofia® Influenza A+B (Quidel, US), which is an immunofluorescence nucleoprotein antigen detection-based test, and Cobas® Liat® (Roche Diagnostics, Spain), an automated multiplex RT-PCR based assay, as described previously (10). Both assays were used according to the user's manual to give results concerning influenza infection within 15 (Sofia®) and 20 min (Cobas® Liat®). Residual volume of all respiratory specimens were kept frozen at primary care centers until they were sent to the laboratory for the definitive influenza confirmation at the end of the season by a commercial, one-step, multiplex, RT-PCR assay (Allplex^TM^, Respiratory Panel Assay, Seegene, South Korea), which is the current routine respiratory virus confirmation technique in the hospital's reference laboratory. Since Sofia® and Cobas® Liat® showed similar and adequate sensitivities and specificities in this patient cohort during the first season (2016–2017) (10), Sofia® was the only test used during the second season and after running the test in primary care, residual volume of specimens was kept frozen and were used for definitive influenza confirmation using the same procedure as the first season. During the third season, Sofia® tests further provided real time results through a cloud system to the reference laboratory. No clinical data were collected during the third season and no confirmation RT-PCR was performed.

Regarding the statistical analysis, antibiotic treatment and use of primary care healthcare services for enrolled patients with a confirmed influenza diagnosis (Group 1), which were obtained through 10 days follow up, were compared with data collected from electronic health records of patients with similar age, geographic area, time period, an attended in other primary care centers. For comparison, 2 groups were established: patients coded J11 (ILI with no influenza virus confirmation; Group 2) and patients coded R50 (fever of other or unknown origin; Group 3). All these codes are included in the ICD-10 2016 classification.

Univariate methods were used to describe the sampling. Comparison of antibiotic consumption and frequency of consultations at the healthcare center was performed by comparative statistics using bivariate analysis (χ2 and Student's *t*-test). A *P* < 0.05 was considered to be significant.

The sensitivity, specificity, positive (PPV) and negative (NPV) predictive values, and inter-rate agreement (Cohen's kappa, k) for both RIDTs in comparison with routine PCR assay were determined using MedCalc® Statistical Software v18.9.1 (MedCalc® bvba, Belgium).

Weekly data provided by the sentinel surveillance network for acute respiratory infections coordinated by the Public Health Agency of Catalonia (Spain), available at: https://canalsalut.gencat.cat/ca/professionals/vigilancia-epidemiologica/pla-dinformacio-de-les-infeccions-respiratories-agudes-a-catalunya-pidirac/index.html#googtrans(ca|en) were compared with data collected in the present study from primary care centers and data collected from the reference hospital.

## Results

During the first season, a total of 189 nasopharyngeal samples were collected, of which 93 (49%) were positive for influenza virus. Some data of this first season have been previously published (10) and are included in this three-season comparison of clinical data, antibiotic use and additional visits. During the first season only influenza A circulated, and a small number of tests was performed in comparison to the following season. During the second and third seasons, both influenza A and B circulated, and the number of nasopharyngeal tests was much higher, 610 tests with 391 (64%) positive and 856 with 443 (52%) positive, respectively.

Study demographics and clinical signs and symptoms during the 2016–2017 and 2017–2018 seasons are summarized in Table 1. The mean age of children with POCT-confirmed influenza virus infection was 31 months for the 2016–2017 season and 37 months for the 2017–2018 season. Patients presented long-lasting clinical signs and symptoms: mean duration of fever >5, cough >8, and rhinorrhoea >8 days. Duration of fever (5 vs. 4 days, *P* < 0.01), cough (8 vs. 7 days, *P* = 0.045), and feeding difficulties (i.e., anorexia; 5 vs. 4 days; *P* = 0.036) were significantly longer in influenza-positive patients during the first season. The same trend was also observed in the second season, in which rhinorrhoea also lasted significantly longer (9 vs. 7 days, *P* < 0.001). During the 2016–2017 season, differences between symptoms and clinical signs from patients with confirmed IAV or IBV could not be calculated because IBV did not circulate. However, during the 2017–2018 season, when both influenza viruses circulated, no clinical differences were observed.

**Table 1:** Study demographics and clinical signs & symptoms of included patients.

	**Season 2016–2017 (*****N*** **=** 189)^*^			**Season 2017–2018 (*****N*** **=** **610)**					
	**IAV (*N* = 91)**	**Influenza negative (*N* = 93)**	***P*^**¥**^**	**Influenza positive (A or B; *N* = 391)**	**Influenza negative (*N* = 219)**	***P***	IAV (*N* = 207)^**^	IBV (*N* = 181)^**^	***P***
Age, months:	31.2	25	0.05	36.9	26	<0.001	33.9	40.4	0.47
mean, (SD; range)	(16.5; 4–60)	(14–60)		(18.7; 2–72)	(18.8; 3–72)		(18.8; 2–72)	(17.9; 3–72)	
Sex: male/female	43/48	41/52	0.61	179/212	128/91	0.003	99/108	80/101	0.001
**Mean duration of symptoms: days (SD)**									
Fever	5.36 (2.16)	4.02 (2.07)	<0.01	5.62 (2.02)	4.37 (2.12)	<0.001	5.63 (2.02)	5.60 (2.04)	0.90
Max. temperature °C	38.9 (0.52)	38.7 (1.14)	0.28	38.8 (0.54)	38.8 (0.59)	0.126	38.9 (0.51)	38.8 (0.57)	0.158
Rhinorrhoea	8.73 (3.74)	7.68 (4.20)	0.79	8.52 (4.07)	7.47 (4.48)	0.005	8.58 (4.23)	8.42 (3.89)	0.713
Cough	8.23 (3.71)	6.99 (4.50)	0.045	7.70 (4.28)	5.94 (4.25)	<0.001	8.05 (4.25)	7.26 (4.30)	0.077
Irritability	3.81 (3.66)	3.20 (2.98)	0.20	3.11 (3.33)	2.71 (3.04)	0.160	3.30 (3.23)	2.90 (3.45)	0.249
Anorexia	5.06 (2.97)	4.12 (3.04)	0.036	4.44 (3.90)	3.40 (3.55)	0.002	4.42 (3.82)	4.48 (4.00)	0.892
Vomiting	0.73 (1.50)	0.73 (1.84)	0.99	0.80 (1.51)	0.69 (1.43)	0.387	0.87 (1.53)	0.71 (1.45)	0.281
Diarrhea	1.02 (2.01)	0.73 (1.81)	0.30	0.76 (1.81)	1.20 (2.65)	0.02	0.79 (1.87)	0.73 (1.74)	0.734
Headache	0.98 (2.00)	0.46 (1.25)	0.037	0.83 (1.75)	0.43 (1.36)	0.005	0.75 (1.61)	0.90 (1.86)	0.428
Myalgia	0.86 (1.73)	0.40 (1.34)	0.046	1.10 (1.96)	0.46 (1.45)	<0.001	1.08 (1.84)	1.12 (2.70)	0.822

* IBV did not circulate during the 2016–2017 season

** *3 patients had a combined A+B infection (not included in this analysis)*;

¥ *P < 0.05 is significant*.

*IAV, influenza A virus; IBV, influenza B virus; N, number of patients*.

Antibiotic consumption data and additional reassessment visits during the 2016–2017 season have already been published (10), except that in this analysis Group 3 only includes patients having a diagnosis of fever (R50) without any microbiologic confirmation (Table 2). For both seasons, influenza-confirmed patients (i.e., Group 1) received fewer antibiotics during the 10 days after influenza diagnosis compared with Group 3 (2016–2017: 4.4% vs. 11.5; 2017–2018: 10.2 vs. 17.2%), but this difference was only statistically significant during the 2017–2018 season (*P* = 0.002). Influenza-confirmed patients in both seasons showed significant lower additional visit rate (2016–2017: 0.19 additional visits per patient; 2017–2018: 0.23) compared to Group 2 (2016–2017:0.48, *P* = 0.001; 2017–2018: 0.49, *P* < 0.001) and Group 3 (2016–2017: 1.01, *P* = 0.001; 2017-2018: 0.80, *P* < 0.001).

**Table 2:** Comparison of antibiotic treatment and additional visits in primary care in the different influenza diagnosis groups.

	Group 1^*^		**Group 2**^**¥**^		**Group 3**^**§**^		***P***^**γ**^** Group 1 vs. Group 2**		***P***^**γ**^** Group 1 vs. Group 3**	
	**2016–2017**	**2017–2018**	**2016–2017**	**2017–2018**	**2016–2017**	**2017–2018**	**2016–2017**	**2017–2018**	**2016–2017**	**2017–2018**
No subjects	91	343	166	769	253	1029				
Age, months:	31.2	35.9	34.0	39.8	27.3	32.1	0.23	0.002	0.07	0.002
mean, (SD; range)	(16.5; 4–60)	(18.6; 2–72)	(16.7; 6–60)	(19.1; 1–72)	(17.4; 6–60)	(20.4; 0–72)				
Antibiotic treatment *n*° (%)	4 (4.4)	35 (10.2)	12 (7.2)	62 (8.1)	29 (11.5)	177 (17.2)	0.38	0.24	0.052	0.002
Additional visits in primary care (SD)	0.19 (0.45)	0.23 (0.47)	0.48 (0.98)	0.49 (1.05)	1.01 (1.57)	0.80 (1.25)	0.001	<0.001	<0.001	<0.001

* *Group 1: Influenza according to results of RT-PCR confirmation after RIDT*.

¥ *Control group 2: Influenza clinical diagnosis (J11)*.

§ *Control group 3: Diagnosis of fever (R50)*.

γ *P < 0.05 is significant*.

*ICD10 2016 coding: J11: Influenza, virus, not identified; R50: Fever of other and unknown origin*.

*Results of first season (N = 91) are based on final PCR, while results of second season (N = 343) are based on Sofia® test*.

Both RIDTs used in our study showed high sensitivities and specificities (Table 3), with excellent agreement with definitive results provided by routine PCR-based assay (gold standard), which is in line with previous manuscripts in pediatric population (11, 12). Sofia® showed high sensitivity and specificity for IAV, particularly for A(H1)pdm09 strains but also for A(H3), specificity for IBV was excellent, but its sensitivity was slightly lower than what has been previously reported in the literature (11).

**Table 3:** Sofia® vs. Allplex®, sensitivity, specificity, PPV, NPV, diagnostic accuracy, and Kappa values.

**Targets**	**No positive specimens**	**Sensitivity % (95% CI)**	**Specificity % (95% CI)**	**PPV % (95% CI)**	**NPV % (95% CI)**	**Diagnostic accuracy % (95% CI)**	**Kappa % (95% CI)**	**Agreement**
Influenza A	191	91.0 (86.2–94.5)	97.0 (94.8–98.4)	94.1 (90.1–96.5)	95.2 (93.0–96.9)	94.9 (92.9–96.5)	0.887 (0.848–0.925)	Excellent
H1pdm09	123	93.2 (87.5–96.8)	83.3 (79.6–86.5)	60.6 (55.6–65.4)	97.8 (95.9–98.8)	85.4 (82.4–88.1)	0.640 (0.574–0.706)	Good
H3	68	87.2 (77.7–93.7)	74.6 (70.7–78.3)	33.5 (29.9–37.4)	97.5 (95.7–98.6)	76.2 (72.7–79.6)	0.367 (0.293–0.441)	Moderate
Influenza B	137	74.4 (67.5–80.6)	98.6 (97.0–99.5)	95.8 (91.1–98.1)	89.9 (87.5–92.0)	91.3 (88.8–93.4)	0.780 (0.724–0.836)	Good

*CI, confidence interval; NPV, negative predictive value; PPV, positive predictive value*.

**Figure 1:**
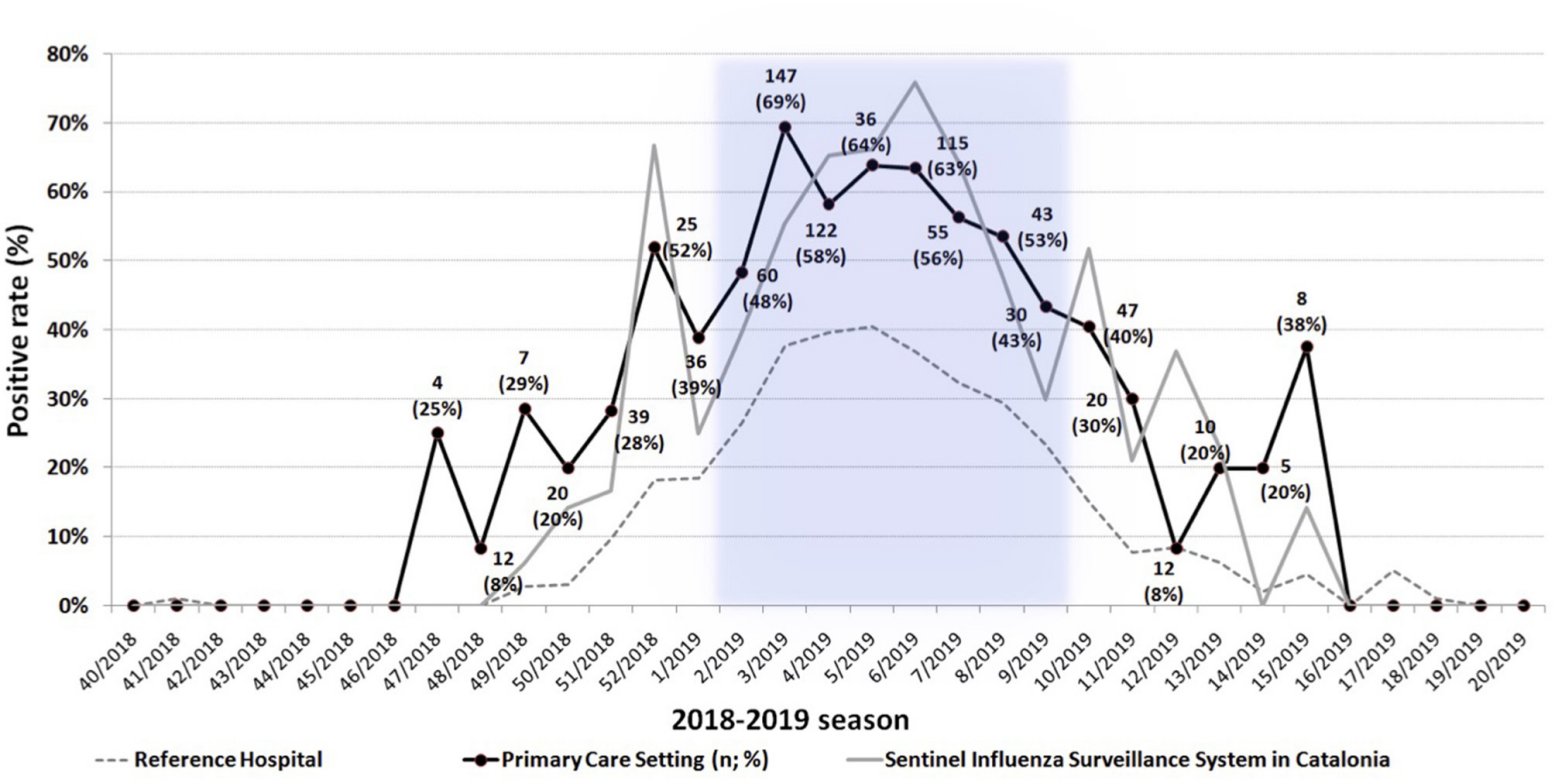
Weekly distribution of positive rate (%) of tested samples (*n*) during the season 2018–2019 collected at the primary care settings, the reference hospital, and sentinel influenza surveillance system. The period of influenza epidemics (more than 110.7 infuenza-like illness cases per 100,000 inhabitants) in Catalonia according to Catalan Public Health Agency is highlighted in blue.

During the third season (2018–2019), weekly distribution of primary care positive RIDTs results were compared to those obtained from (i) the reference hospital and (ii) the Catalan sentinel influenza surveillance system. The first influenza-confirmed cases from both primary care and reference hospital were detected between 1 and 2 weeks earlier than those of the surveillance system during the initial steps of the seasonal influenza epidemic (Figure 1).

## Discussion

Influenza in healthy children can be associated with severe morbidity and mortality (6) although in most cases it is a self-limited and usually uncomplicated disease that needs only symptomatic treatment. Hence, it is crucial to have a microbiological diagnosis in few minutes to confirm the respiratory infection due to influenza virus in the primary care setting, in those cases in which the clinician cannot make an accurate clinical diagnosis, usually infants and young children which may present with high fever as the only symptom, or when pre-existing health condition may warrant antiviral treatment. In recent years, sensitive and specific RIDTs have been commercialized for such purpose, either based on antigen (Sofia®) or nucleic acid detection (Cobas® Liat®) (12–14). The latter is often more sensitive, but its higher cost can represent an economic burden to the healthcare system in many countries, a fact that may hinder its use in daily clinical practice. In our previous manuscript of the first season (10), the molecular RIDT (Cobas® Liat®) showed a slightly higher sensitivity than the immunofluorescent assay (Sofia®), and thus the overall accuracy of diagnosis improved, although the difference was not statistically significant. On this basis we considered that the immunofluorescence, nucleoprotein, antigen detection-based test (Sofia®), was adequate for pediatric primary care patients because their viral load is very high in nasopharynx, especially at the onset of the symptoms and therefore we used it as the only test during the following two seasons.

Based on our experience, RIDTs are an excellent tool for primary care pediatricians to correctly confirm influenza infection in infants and young children presenting non-specific symptoms. In the US, RIDTs aim to identify patients who can benefit of oseltamivir treatment started within the first 48 h after the onset of symptoms. However, in other countries, where antiviral treatment is not an usual option in primary care, the use of RIDTs have other purposes such as: (i) minimizing the use of antibiotics, (ii) making an aetiological diagnosis that reassures the patient, and (iii) explaining the possible evolution of the disease, thus enhancing the trust of families in their healthcare provider.

In our study, as shown in Table 2, patients with a clinical diagnosis of influenza-like illness (Group 2) or diagnosed initially as fever (Group 3) doubled or tripled the number of additional consultations in comparison with RIDT-confirmed patients (Group 1). Prolonged symptoms and uncertainty about their cause are often the reasons for families to revisit their pediatrician or attend the emergency room. On the other hand, influenza-confirmed patients had less additional visits in primary care because the expected evolution of disease can be better explained to the family based on the certainty of an objective test. The additional visits for the same process make an important difference as the healthcare system is usually under pressure due to the high number of consultations during the influenza season (15, 16).

Antibiotic prescriptions were also reduced when patients had a confirmed RIDT confirmation of influenza, compared to those having a clinical diagnosis without microbiologic confirmation, although the differences were statistically significant only for the 2017–2018 season. Our results are in line with data published by other groups, like Tillekeratne et al. (17), who showed a 20% decrease in the antibiotic prescription with the use of an POCT for influenza confirmation, and Jennings et al. (18), who showed a five-fold increase of antibiotic prescriptions in clinical diagnosed patients. Egilmezer et al. (19) recently published a systematic review of the impact of diagnosing influenza with a POCT in patients with acute respiratory tract infection. The main findings were that a positive POCT significantly increased the proper use of antivirals and decreased antibiotic prescriptions. Unfortunately, the number of papers addressing prospectively the impact of influenza POCTs on antibiotic treatment in pediatric primary care were only two out of the 30 papers included in this systematic review (19). These papers confirm that decision-making process of an antibiotic prescription is vastly influenced by an accurate diagnosis (17, 20).

One of the strengths of our study is that our data are based on the use of a POCT in pediatric primary care, while most of the published data come from emergency services or hospitalized patient cohorts (19). A limitation of our study was that the data from clinically diagnosed patients without influenza confirmation or patients with fever (Groups 2 and 3) were extracted from the EHR of similar patients visited at other health care centers within the same geographical area, and although the system is capable of detecting any antibiotic prescription or visit to the primary health care system, these data could be higher due to over-the-counter purchased antibiotics or health care visits at private pediatricians or other health care providers. Data collected during the 10-days follow-up of RIDT-confirmed patients (Group 1) are more exact as they included any antibiotic prescription and health care visit. The differences between groups mentioned in this paper are therefore a bottom line and could be higher.

One of the results to be highlighted of this study is the value of real-time transmission of RIDTs results from pediatric primary care patients for the surveillance of influenza viruses for the whole community, as this system may detect early circulating strains. Still more, sentinel surveillance system data need usually a week from collection to publication whilst RIDTs and hospital results are daily real-time reported without any delay. Therefore, RIDTs data may be complementary to data collected traditionally through the sentinel surveillance systems.

In summary, the use of RIDTs has a great potential in primary care specially in infants and young children, allowing a better management of these patients, less additional consultations in primary care reducing therefore the burden of disease on the health care system. The results of the RIDTs done in primary care provide a good source of real-time surveillance data when a cloud system connected to the reference hospital is available.

## Data Availability Statement

The datasets generated for this study will not be made publicly available. Confidentiality of patient data.

## Ethics Statement

The studies involving human participants were reviewed and approved by Idiap Jordi Gol. Evaluated 28/09/2016 and approved code number P16/135. Written informed consent to participate in this study was provided by the participants' legal guardian/next of kin.

## Author Contributions

DE, MV, CR, TP, and AA conceived and designed the study. MV, SI, JF, LL, GR, LS, PM, MP, MªF, and EH recruited the patients performed point-of-care tests, preserved respiratory samples prior to the shipment to the reference hospital, and collected the clinical data. CA performed the laboratory influenza confirmation test at the reference hospital. DE, CA, and AA analyzed and discussed results, drafted the manuscript, tables, and figures. CR and TP aided in interpreting the results. DE coordinated the execution of this study. All authors discussed the results and commented on the manuscript.

## Conflict of Interest

The authors declare that the research was conducted in the absence of any commercial or financial relationships that could be construed as a potential conflict of interest.
